# A Novel Hypovirus Species From Xylariaceae Fungi Infecting Avocado

**DOI:** 10.3389/fmicb.2018.00778

**Published:** 2018-05-08

**Authors:** Leonardo Velasco, Isabel Arjona-Girona, María T. Ariza-Fernández, Enrico Cretazzo, Carlos López-Herrera

**Affiliations:** ^1^Instituto Andaluz de Investigación y Formación Agraria, Málaga, Spain; ^2^Instituto de Agricultura Sostenible, Consejo Superior de Investigaciones Científicas, Córdoba, Spain

**Keywords:** hypovirus, *Entoleuca*, *Rosellinia necatrix*, avocado, population genetic analysis, biocontrol

## Abstract

The white rot root disease caused by *Rosellinia necatrix* is a major concern for avocado cultivation in Spain. Healthy escapes of avocado trees surrounded by diseased trees prompted us to hypothesize the presence of hypovirulent *R. necatrix* due to mycovirus infections. Recently, we reported the presence of another fungal species, *Entoleuca* sp., belonging to the *Xylariaceae*, that was also found in healthy avocado trees and frequently co-infecting the same roots than *R. necatrix*. We investigated the presence of mycoviruses that might explain the hypovirulence. For that, we performed deep sequencing of dsRNAs from two isolates of *Entoleuca* sp. that revealed the simultaneous infection of several mycoviruses, not described previously. In this work, we report a new member of the *Hypoviridae*, tentatively named Entoleuca hypovirus 1 (EnHV1). The complete genome sequence was obtained for two EnHV1 strains, which lengths resulted to be 14,958 and 14,984 nt, respectively, excluding the poly(A) tails. The genome shows two ORFs separated by a 32-nt inter-ORF, and both 5′- and 3′-UTRs longer than any other hypovirus reported to date. The analysis of virus-derived siRNA populations obtained from *Entoleuca* sp. demonstrated antiviral silencing activity in this fungus. We screened a collection of *Entoleuca* sp. and *R. necatrix* isolates and found that EnHV1 was present in both fungal species. A genetic population analysis of EnHV1 strains revealed the presence of two main clades, each of them including members from both *Entoleuca* sp. and *R. necatrix*, which suggests intra- and interspecific virus transmission in the field. Several attempts failed to cure *Entoleuca* sp. from EnHV1. However, all *Entoleuca* sp. isolates collected from avocado, whether harboring the virus or not, showed hypovirulence. Conversely, all *R. necatrix* isolates were pathogenic to that crop, regardless of being infected by EnHV1.

## Introduction

Collectively, fungi and fungal-like organisms (FLOs), such as oomycetes, cause more plant diseases than any other group of plant pests with more than 8,000 species that have been shown to be phytopathogenic. To understand the staggering impact of fungi on plant health, food loss, and human nutrition, examples such as potato blight or ergot of rye are enough. Therefore, the control of phytopathogenic fungi (physical, chemical, agronomic, or biological) has represented for a long time a basic topic for science and technology. In the 1970s, mycoviruses were discovered in plant filamentous fungal pathogens such as *Helminthosporium victoriae* (Sanderlin and Ghabrial, 1978) and *Cryphonectria parasitica* (Van Alfen et al., 1975). Since then, the interest of scientific community on mycoviruses has been rising as they are able to induce hypovirulence and lower mycelial growth rate and conidial production, and consequently to be used in disease control. However, comprehensive knowledge on taxonomy, ecology, and evolution of many mycoviruses hosted by harmful fungi (i.e., *R. necatrix* and *Fusarium* sp.) has been achieved only recently, due to the advances in deep-sequencing technologies. Reviews on fungal mycoviruses are provided by Xie and Jiang (2014) and Ghabrial et al. (2015).

Mycoviruses are widespread in all major taxa of fungi and are grouped into several families. Members of *Hypoviridae* have positive ssRNA genomes, ranging from 9 to 14 kb, which do not encode for structural proteins and produce dsRNAs in their hosts. The four types of species are Cryphonectria hypovirus 1–4 (CHV1, CHV2, CHV3, and CHV4) (Suzuki et al., 2018). CHV1 is largely the most extensively studied mycovirus for the *C. parasitica* hypovirulence phenomena on *Castanea* and *Quercus* species (Heiniger and Rigling, 1994), with at least five subtypes identified and tested for chestnut blight control. In addition, several other species have been reported including Fusarium graminearum hypovirus 1 and 2 (FgHV1 and FgHV2), Phomopsis longicolla hypovirus 1, Sclerotinia sclerotiorum hypovirus 1 and 2 (SsHV1 and SsHV2), and Valsa ceratosperma hypovirus 1 (VcHV1) (Xie et al., 2011; Yaegashi et al., 2012; Khalifa and Pearson, 2013; Wang et al., 2013; Hu et al., 2014; Koloniuk et al., 2014; Li et al., 2015). It is assumed that family *Hypoviridae* is formed by a sole genus namely *Hypovirus*, although three genera have been proposed recently (Alphahypovirus, Betahypovirus, and Gammahypovirus) depending on the presence of either one or two ORFs, in addition to phylogeny and analyses of the putatively encoded proteins (Li et al., 2015).

The causal agent of white rot root in avocado, *R. necatrix*, represents a major concern for this crop in Spain (López-Herrera, 1998). Several strategies are underway aiming to control this disease. Among them, virocontrol is considered a plausible tool and the search for mycoviruses that may limit fungus growth has been initiated. Instead, *Entoleuca* sp. has not yet shown any pathogenicity on avocado, although a member of the genus *Entoleuca* (*E. mammata*) is pathogenic for several genera of hardwood trees (Ostry and Anderson, 2009). *Entoleuca* sp. and *R. necatrix* are not easily distinguishable morphologically, and consequently molecular methods are needed for correct identification (Bahl et al., 2005). These fungi appear to compete for colonization of avocado roots. Therefore, *Entoleuca* sp. has been recently proposed as a biocontrol agent of white root rot in avocado (Arjona-Girona and López-Herrera, 2018). On the other hand, we found that *Entoleuca* sp. and *R. necatrix* share a consistent number of infecting mycoviruses belonging to different families that are known to be hosted in other fungal species (Velasco et al., unpublished). Therefore, a natural spreading in the field from one fungus to another can be supposed, suggesting the possibility to use these mycoviruses as alternative biocontrol sources for white root rot in avocado.

In this work, we report a novel hypovirus species detected in *R. necatrix* and *Entoleuca* sp. from avocado orchards in Malaga province (Southern Spain) and tentatively named Entoleuca hypovirus 1 (EnHV1). An RT-PCR test allowed the detection of this virus in a set of *Entoleuca* sp. and *R. necatrix* isolates collected from avocado roots. A population study of EnHV1 strains showed two main clades with members from both fungal species. Finally, we investigated the interaction of EnHV1 with its fungal host.

## Materials and Methods

### Fungal Isolates Collection and *Entoleuca* sp. Determination

Thirty-nine fungal isolates (20 *Entoleuca* sp. and 19 *R. necatrix*) were collected from avocado escape trees of four orchards located in the Malaga province (Spain) spanning along a track of 35 km (Arjona-Girona and López-Herrera, 2018). These plants were surrounded by white root rot symptomatic avocado trees. For each isolate, mycelia were grown on PDA media and used for subsequent analyses. Differentiation between *R. necatrix* and *Entoleuca* sp. was based on sequencing of the genomic ITS region amplified with ITS4 and ITS5 primers (Arjona-Girona and López-Herrera, 2018).

### DsRNA Extractions and High-Throughput Sequencing

For each sample, 1 g of fungal tissue was grown for 10 days on cellophane membrane-covered PDA Petri plates. Then, the dsRNAs were extracted using CF-11 cellulose as described by Morris and Doods (1979). Quality and yield were determined using the NanoDrop ND-1000 Spectrophotometer (NanoDrop Technologies). DsRNA band sizes were examined in 1% agarose gels after RedSafe^TM^ staining under UV light. After extraction, dsRNAs were eluted in 50 μl RNase-free water and stored at -70°C until further use. Five to ten micrograms of dsRNA of the *Entoleuca* sp. isolates E97-14 and E112-4 was used for high-throughput sequencing. Prior to be processed, the dsRNAs were denatured at 95°C for 10 min and immediately cooled on ice. Next, RNA fragmentation and cDNA synthesis were carried out. Libraries were prepared following procedures developed at the CRG Genomic Unit (Barcelona, Spain). Illumina sequencing was performed using the Hiseq2000 for 50 nt reads. After removing adaptors and low-quality reads, contigs were generated by *de novo* assembly using the algorithm Velvet v.12.08 (k-mer = 33) at the SCBI Picasso Supercomputing server (Malaga, Spain). Contig data sets were subjected to BLASTN and BLASTX in the NCBI GenBank through the software Geneious version 7.1.9 (Biomatters). Draft consensuses of the tentatively named Entoleuca hypovirus 1 (EnHV1) were obtained by the assembling of contigs from strains 97-14 and 112-4. Sanger sequencing was used to check the correctness of the consensus sequences by obtaining amplicons along the genome of the virus and the 5′- and 3′-ends were determined (see below).

### 5′- and 3′-Ends Determination of the EnHV1 Genomes

For 5′- and 3′-ends determination, the protocol developed by Potgieter et al. (2009) was performed with some modifications. The oligo ligation reaction included the following steps: (a) 200 ng of purified dsRNA was added to a mixture containing 250 ng of PC3-T7 loop primer, T4 Ligase Buffer (Ambion), 0.1% BSA (Sigma), 10% DMSO (Sigma), 20% PEG6000 (AppliChem), 20 units of Ribolock RNase inhibitor (Thermo Fisher), and 40 units of T4 RNA ligase (Ambion). (b) Ligation components were incubated at 37°C for 6 h and then at 18°C descending down to 12°C for overnight. (c) Primer-ligated dsRNAs were diluted in 200 μl DEPC H_2_O and purified from primer excess by precipitation with 8 M LiCl and ethanol. (d) Purified primer-ligated dsRNAs were denatured at 98°C for 2 min in the presence of 1 M betaine and 2.5% DMSO and then quenched on ice for 5 min. Finally, the synthesis of cDNA was performed using the MultiScribe Reverse Transcriptase (Applied Biosystems) in the presence of dNTPs and RNase inhibitor for 1 h at 42°C. The cDNA was used as template for 5′- and 3′-end amplifications using specific primers (not shown) as forward and the PC2 primer (5′-CCGAATTCCCGGGATCC-3′) as reverse. The amplicons obtained were cloned and sequenced.

### Small RNA Obtention, Library Construction, and Sequencing

Total RNA from *Entoleuca* sp. isolate E97-14 was extracted using the Spectrum Plant RNA Kit (Sigma). Next, the small RNA fraction within the 18–50 nt range was eluted from polyacrylamide gels and the corresponding cDNA libraries were prepared. Subsequent sequencing was performed at the Illumina NextSeq 550 platform, available at the Supercomputing and Bioinnovation Center (SCBI, University of Malaga, Spain) and data were processed as described above. Contigs were assembled using Velvet (k-mer = 17). Reads and contigs were aligned to EnHV1 genome in Geneious.

### Sequence and Phylogenetic Analysis

Analysis of regulatory motifs in the 5′- and 3′-UTR genome regions of the EnHV1 was accomplished using the RegRNA 2.0 online server^1^ (Chang et al., 2013). Domain prediction was performed at the NCBI CDD or by architecture domain search (Marchler-Bauer et al., 2013) and the TMHMM transmembrane prediction tool version 0.9 available in Geneious. Secondary structures possibly involved in the coupled termination-reinitiation of virus translation were searched by DotKnot (Sperschneider and Datta, 2010) in the inter-ORFs region and in the downstream proximity of the start codon of downstream ORF. The deduced amino acid (aa) sequence of the EnHV1 replicase protein was aligned to a set of sequences retrieved from the GenBank by the MAFFT algorithm (Katoh et al., 2002) in Geneious. In order to generate a maximum-likelihood (ML) phylogenetic tree including EnHV1 and other representative members of family *Hypoviridae*, the replicase polyprotein sequences were analyzed and the best-fit models were tested on ProtTest version 3.3 (Darriba et al., 2011). The final tree was obtained using the PHYML plugin (Guindon et al., 2010) in Geneious.

### RT-PCR Detection of EnHV1 in Other *Entoleuca* sp. and *R. necatrix* Isolates

The primer pair HYPRD-F (5′-GCGTACCAGGAACCAGAGTATAG-3′) and HYPRD-R (5′-AGGGGCGAGTATTATAACACGTC-3′) based on the consensus sequence of ORF2 (positions 10,872 and 11,555 from strain 97-14, see the section “Results”) was used for the determination of EnHV1 in the 37 isolate collection. DsRNAs were obtained using the dsRNA extraction kit (Intron). The synthesis of cDNA by reverse transcription was performed with 200 ng of dsRNA, 10 pmol of random primers, and the MultiScribe RT (Applied Biosystems). PCR reactions were carried out with Platinum Taq DNA polymerase (Invitrogen) using 50 pmol of each primer in the following conditions: an initial 5 min denaturation at 94°C, then 40 cycles of 30 s at 95°C, 30 s at 55°C, and 1 min at 72°C, and a 5 min final extension at 72°C.

### Genetic Variability of EnHV1 Strains From *Entoleuca* sp. and *R. necatrix*

Multiple sequence alignments were performed at the nt and aa levels using the MAFFT algorithm (Katoh et al., 2002) as implemented in Geneious for the partial replicase genomic region of 21 EnHV1 strains, chosen among RT-PCR positive isolates (see the section “Results”). The best nucleotide substitution model for each genomic region was determined by JMODELTEST using AIC_c_ (Santorum et al., 2014). A ML phylogenetic tree was inferred analyzing the partial replicase genomic region with PHYML plugin available in Geneious under the HKY85 + I model with four gamma categories (Hasegawa et al., 1985), as it was estimated to be the most appropriate substitution model.

The nucleotide genetic diversity (*d*) and synonymous (*d*S) and non-synonymous (*d*NS) substitutions at codons were calculated using maximum composite likelihood (MCL) algorithms and Pamilo, Bianchi, and Li with 1,000 bootstrap replicates implemented in MEGA version 7 (Kumar et al., 2016). Also, the *d*NS/*d*S ratio was calculated in order to determine the selection pressure. A *d*NS/*d*S < 1 is generally considered as indicative of a negative or purifying selection. The role of natural selection in shaping the EnHV1 population was evaluated by testing the mutation neutrality hypothesis using Tajima’s *D* and Fu and Li’s statistical tests implemented in program DNASP version 5.10 (Librado and Rozas, 2009). Tajima’s *D* test is based on the differences between the number of segregating sites and the average number of nucleotide differences (Tajima, 1989). Fu and Li’s *D* test is based on the differences between the number of singletons (mutations appearing only once among the sequences) and the total number of mutations. Finally, Fu and Li’s *F*^∗^ test based on the differences between the number of singletons and the average number of nucleotide differences between pairs of sequences (Fu and Li, 1993).

In order to identify the aas under selective pressure across the deduced protein sequences, differences between synonymous and nonsynonymous substitutions were calculated for each aa position in the alignment using several comparison methods: the single-likelihood ancestor counting (SLAC), the fixed-effects likelihood (FEL), and the internal fixed-effects likelihood (IFEL) (Kosakovsky Pond and Frost, 2005) available on the Datamonkey server^2^. Additional parameters were also estimated such as the nucleotide polymorphism (p: average estimate of nucleotide differences between two sequences randomly in a population), the statistic Θ, and the number of segregating sites (*S*) (Watterson, 1975).

### Recombination Analysis

The nucleotide sequences of the replicase genomic region were analyzed to determine possible recombination events using the programs GARD (Kosakovsky Pond et al., 2006) and RDP4 v. 4.94 containing the recombination-detecting algorithms GENECONV, BOOTSCAN, MAXCHI, CHIMERA, SISCAN, 3SEQ, LARD, RDP, and PHYLPRO (Martin et al., 2015).

### Pathogenicity Test on Avocado Plants

Experiments were performed for both EnHV1-infected and not infected *Entoleuca* sp. or *R. necatrix* isolates in order to test the pathogenicity of these strains on avocado. For that, 4-month-old avocado plants grown in 3.5 l pots with Laura substrate (60% peat, 20% coconut fiber, and 20% perlite) were initially inoculated with wheat seeds (Sztejnberg and Madar, 1980) that had been previously colonized by isolates with or without EnHV1 infection (as determined by RT-PCR, see below), at the rate of 6 g/l substrate. Additional plants that were either inoculated only with pathogenic Rn400 *R. necatrix* or not inoculated at all were used as positive and negative controls, respectively. Eight plants per isolate were used. Four weeks later, the pathogenicity of the isolates was evaluated.

### Attempts to Eliminate EnHV1 From *Entoleuca* sp. Isolates E97-14 and E112-4 by Hyphal Tipping and Sensitive Detection by RT-qPCR

For both E97-14 and E112-4 isolates, mycelial plugs were taken from PDA and put onto a limited nutrient medium, oatmeal agar (OA). Plates were incubated in the dark at 28°C and when mycelia developed enough, hyphal tips were cut and transferred to other four OA plate sets (five replicas each) containing: (a) 300 μg/ml of cycloheximide (Sigma), (b) 100 μg/ml of ribavirin (Sigma), (c) 75 μg/ml of ribavirin + 150 μg/ml of cycloheximide, and (d) 100 μg/ml of ribavirin + 300 μg/ml of cycloheximide, respectively. Four passages more were performed for each concentration set starting from hyphal tips of the previous step. Finally, from the last tipping steps, three mycelial plugs each (one per plate) were taken and grown on PDA plates carrying a top cellophane film and used for virus detection.

In order to check EnHV1 elimination using a sensitive method, RT-qPCRs were performed in white 96-well PCR plates using a Bio-Rad iQ5 thermal cycler. For that, we designed a primer pair based on a conserved region within the ORF2 of EnHV1: QHYP-F (5′-CACGCAGATCATTGATGCCG-3′) and QHYP-R (5′-GGTTTTTCACCTGTTCCGGC-3′). In order to have an endogenous control for RNA extraction and reverse transcription efficiency, an additional primer pair was designed derived from the 5.8S ribosomal RNA consensus sequences of *Entoleuca* sp. (Accession Nos. KY009713-KY009732) and *R. necatrix* (Accession No. AB017657): RR58S-F (5′-ATCGATGAAGAACGCAGCG-3′) and RR58S-R (5′-AATGACGCTCGAACAGGCAT-3′). Mycelial RNAs were extracted using Trisure (Bioline) following the protocol developed by Schumann et al. (2013) with an additional chloroform extraction step followed by DNase I (Sigma) treatment. The cDNAs were obtained using the High-Capacity cDNA Reverse Transcription Kit (Applied Biosystems) according to manufacturers’ instructions. Each reaction (20 μl final volume), in duplicate, contained 400 ng of cDNA, 10 μl of KAPA SYBR Green qPCR mix (KAPA Biosystems), and 500 nM of each specific primer. Specificity of the amplicons obtained was checked with the Bio-Rad Optical System Software v.2.1, by means of melting-curve analyses (60 s at 95°C and 60 s at 55°C), followed by fluorescence measurements (from 55–95°C, with increments by 0.5°C).

## Results

### Multiple Virus Infection in *Entoleuca* sp. Isolates

After BLASTn and BLASTx submission, the contigs generated from the sequence pool showed homologies to a set of putative fungal and plant viruses, including tentative members of the *Endornaviridae, Hypoviridae, Partitiviridae, Ourmiaviridae, Megabirnaviridae*, among other virus species (Velasco et al., unpublished). In this work, we describe the novel hypovirus species detected in *Entoleuca* sp. and *R. necatrix* from avocado trees in Spain.

### Genomic Organization of Entoleuca Hypovirus 1

The complete sequences of EnHV1 from strains 97-14 and 112-4 (GenBank accession Nos. MF536690 and MF536691, respectively) show slightly different lengths, 14,958 and 14,984 nt, excluding the poly(A) tails. EnHV1 97-14 was chosen as a reference in this work. A 5′-UTR of 945 nt the longest 5′-UTR among hypoviruses reported to date precedes two predicted ORFs. No similarities with other hypovirus sequences were found in the 5′-UTRs. Five upstream mini-ORFs (uORFs) could be detected. In addition, three Internal Ribosomal Entry Sites (IRES) were detected using RegRNA finder (Le and Maizel, 1997). The 5′-UTR in EnHV1 strain 112-14 showed the same motifs in equivalent positions.

**FIGURE 1:**
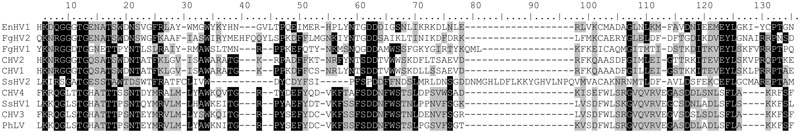
Alignment of the homologous regions corresponding to the putative RNA-dependent RNA polymerase site (superfamily cd01699) in several hypoviruses. Virus abbreviations as in **Figure 3**.

**FIGURE 2:**
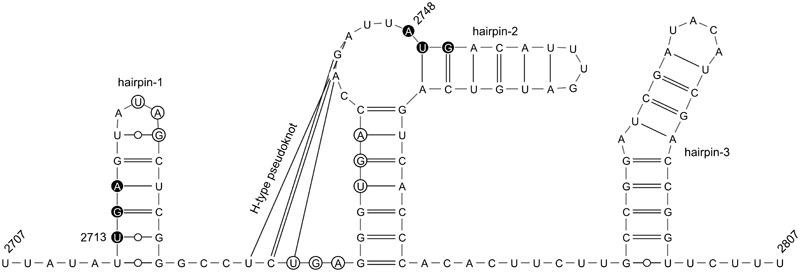
Secondary RNA structure of the inter-ORF region of EnHV1 strain 97-14 was predicted by DotKnot and then drawn by VARNA 3.93 software (Darty et al., 2009). Stop codon for ORF1 and start codon for ORF2 are represented as filled circles and inter-ORF stop codons as empty circles.

EnHV1 ORF1 consists of 1,770 nt starting at position 946 from the 5′-end (950 in the case of 112-4). It encodes for a putative protein of 589 aas showing a predicted molecular mass of 64.5 kDa. No correspondences with identified known domains were found both in the NCBI’s conserved domain database (CDD) and by architecture domain search (Marchler-Bauer et al., 2013). No coiled-coils domains were predicted in the structure according to the EMBnet COILS server (Lupas et al., 1991). In addition, no transmembrane regions were predicted by the TMHMM transmembrane algorithm. ORF2 consists of 10,920 nt starting at position 2,728 from the 5′-end (position 2,752 in the case of strain 112-4). It encodes for a (poly) protein of 3,639 aas and a molecular mass of 413.4 kDa. NCBI’s CDD search allowed the identification between positions 11,421 and 12,240 (aa 2,892–3,178) of a domain corresponding to the DEAD-like helicase superfamily, including the ATP-binding site, the Mg^++^-binding site, a P-loop containing nucleoside triphosphate hydrolases site, and the nucleotide-binding region. No additional domains were found in the ORF2, such as a papain-like protease that is frequent in hypoviruses. Although not matching to the NCBI’s CDD, a region resembling an RNA-dependent RNA polymerase site was detected between positions 9,612–9,934 (aa 2,295–2,399) of the consensus sequence (**Figure 1**). Eight transmembrane regions were predicted according to TMHMM analysis. Therefore, this ORF2 probably encodes for the viral replicase. Given that ORF1 and ORF2 are not overlapping, an inter-ORF of 32 nt is present. Analysis of the inter-ORF RNA secondary structure revealed the presence of a predicted H-type pseudoknot which base pairs form two opposite stem-loops 2 nt before the ORF2 start codon (**Figure 2**). Also, a predicted hairpin structure includes the ORF1 stop codon, while two other hairpins are predicted immediately and 29 nt downstream the ORF2 start codon, respectively (**Figure 2**). The 3′-UTR consists of 1,279 nt, the longest among the hypoviruses described so far. Motif search did not allow the identification of any functional element in this region. No significant similarities were found by comparing with other virus sequences.

### Genetic Allocation of EnHV1 in the Family *Hypoviridae*, Genus Alphahypovirus

**FIGURE 3:**
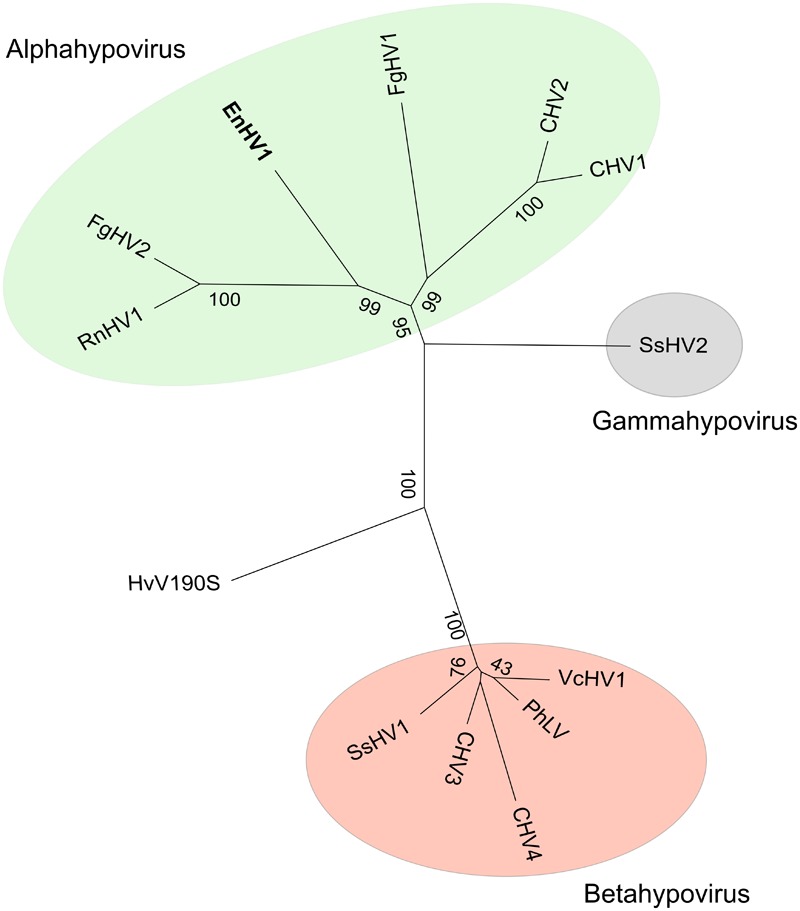
ML phylogenetic tree based on the aa sequences of the viral replicases of a set of hypoviruses. The tree was generated with the WAG+F model of aa substitution. Bootstrap values of consensus support are indicated on branch nodes. The genome of Helminthosporium victoriae virus 190S (HvV190S), a victorivirus, was used as out-group (Accession No. U41345). Two separate clades are evident. EnHV1, Entoleuca hypovirus 1 (MF536690); CHV1, Cryphonectria hypovirus 1 (M57938); CHV2, Cryphonectria hypovirus 2 (L29010); CHV3, Cryphonectria hypovirus 3 (AF188515); CHV4, Cryphonectria hypovirus 4 (AY307099); FgHV1, Fusarium graminearum hypovirus 1 (KC330231); FgHV2, Fusarium graminearum hypovirus 2 (KP208178); PhLV, Phomopsis longicolla hypovirus (KF537784); RnHV1, Rosellinia necatrix hypovirus 1 (NC_036590); SsHV1, Sclerotinia sclerotiorum hypovirus 1 (JF781304); SsHV2, Sclerotinia sclerotiorum hypovirus 2 (KF898354); and VcHV1, Valsa ceratosperma hypovirus 1 (AB690372).

EnHV1 showed similar genome organization than other hypoviruses. An ML tree based on the aa sequences of the viral replicases locates EnHV1 in the same branch as FgHV2 and proximal to CHV1 (**Figure 3**), which have been proposed as members of the tentative genus Alphahypovirus (Li et al., 2015). The EnHV1 replicase polyprotein showed the highest aa identities with members of this genus: FgHV2 (18.3%), CHV1 (17.9%), CHV2 (17.2%), FgHV1 (15.2%), and SsHV2 (9.4%). Regarding the putative RdRp domain in the replicase, the aa identities between EnHV1 and the other viruses increased to: FgHV2 (29.11%), CHV1 (27.0%), CHV2 (24.5%), FgHV1 (23.7%), and SsHV2 (20.9%).

### Comparison Between the Two Complete Genomes of EnHV1

**FIGURE 4:**
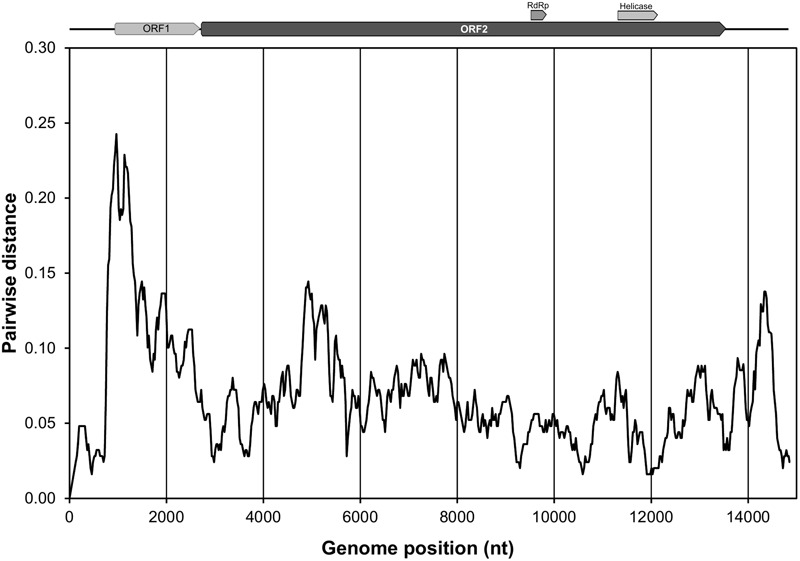
Plot of the pairwise genetic distances along the genomes of Entoleuca hypovirus 1 strains 97-14 and 112-4. Genomes were aligned and mean distances were calculated for overlapping windows of 250 nt shifted by 25 nt and plotted against the midpoint of the windows. A schematic view of the EnHV1 genome with the predicted ORF1 (light filled arrow) and ORF2 (dark filled arrow), including two hypothetical domains, is presented for reference. The plot was generated using SSE v. 1.2 (Simmonds, 2012).

Pairwise nucleotide identity between 97-14 and 112-4 EnHV1 strains resulted 95.2%. Protein identity was 84.6 and 96.8% for ORF1 and ORF2, respectively. A nucleotide distance plot showed that differences were higher in the 5′-half of the genomes, corresponding to the ORF1 (**Figure 4**). Interestingly, the highest identity was observed in the 5′-UTR, where the first 164 nt was 100% identical, and in the rest of the 5′-UTR sequence identity decreased to 97%. This high sequence conservation was not observed at the 3′-UTR that showed only 91.0% nucleotide identity. Besides, motifs identified in the 5′-UTR (uORFs and IRES) and the 3′-UTR (SECIS) were conserved in both strains.

### Analysis of Virus-Derived Small Interfering RNAs Derived From EnHV1

**FIGURE 5:**
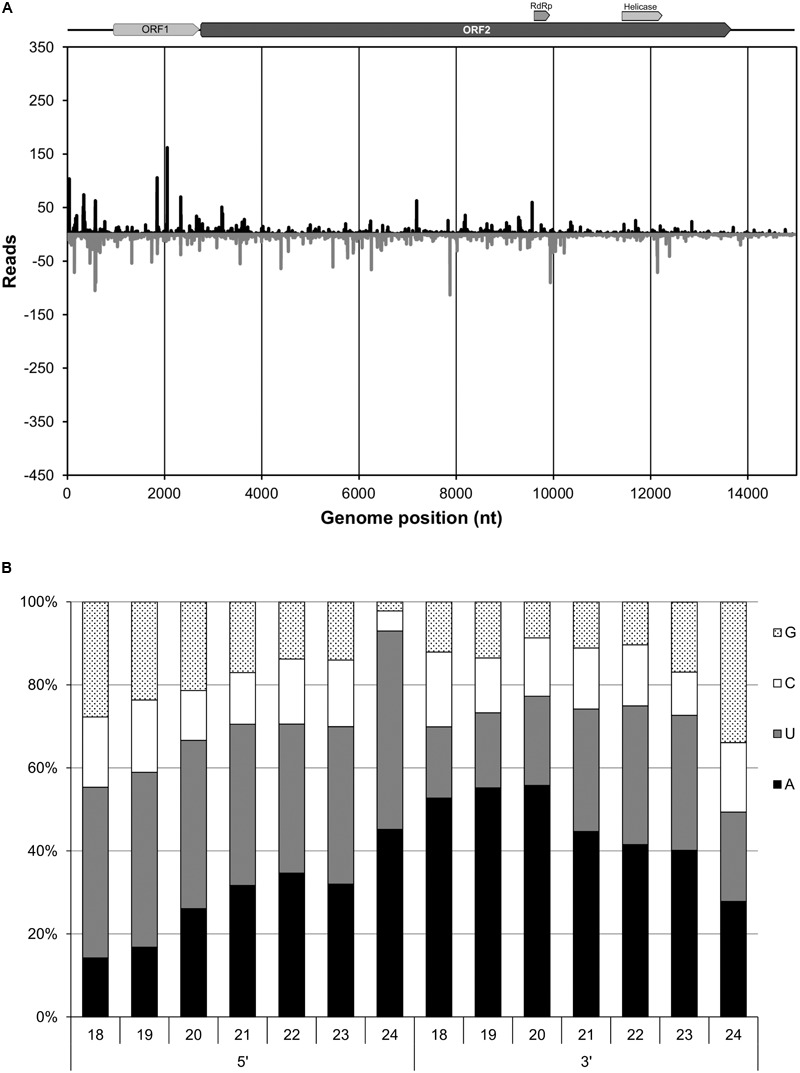
Profile distribution of virus small interfering RNAs (vsiRNAs) of the 21-nt class with respect to the Entoleuca hypovirus 1 complete genome. Peaks above and below the *x*-axis stand for sense and antisense vsiRNA orientations, respectively. For analysis, vsiRNA populations were aligned to the indexed EnHV1 97-14 genome using Geneious and the BAM alignment file produced was processed using MISIS-2 (Seguin et al., 2016). Separation between marks in the *x*-axis represents 1,000 nucleotides **(A)**. Percentages of nucleotide species at the 5′- and 3′-ends of redundant vsiRNAs derived from EnHV1 strain 97-14 according to the size of the reads **(B)**.

Accumulation of vsiRNAs is a distinctive characteristic of RNA silencing-based antiviral defense mechanisms and its analysis allowed investigating the interaction between *Entoleuca* sp. and EnHV1. A total of 8.55 million small RNA sequencing reads from 18 to 45 nt length was obtained from E97-14. Reads over 25 nt length matched poorly to the EnHV1 genome. Then, we restricted the reads to the range from 18 to 24 nt length (3.70 million reads). The 21-nt small RNA class population was prevalent followed by the 20 and 22-nt classes (**Supplementary Figure S1**). Within the population of 1.02 million 21-nt class reads, 13,220 vsiRNAs aligned to the EnHV1 genomic sequence in sense and antisense directions (**Figure 5A**). Negative-sense 21-nt vsiRNAs (54.3%) prevailed over positive sense ones. Similar profiles and proportions were derived after aligning the 20-nt and the 22-nt classes (not shown). On the other hand, determining the nucleotide frequencies at the 5′- and 3′-positions of the vsiRNA species provides valuable information of the specific silencing activities of the host. There was a bias toward A and U for those positions in the redundant 18–24 nt classes of EnHV-derived vsiRNAs (49.3–93%) (**Figure 5B**). Finally, 13 out of 547 contigs obtained from the 18- to 24-nt classes matched to EnHV1.

### Detection of EnHV1 in Other *Entoleuca* sp. and *R. necatrix* Isolates

**FIGURE 6:**
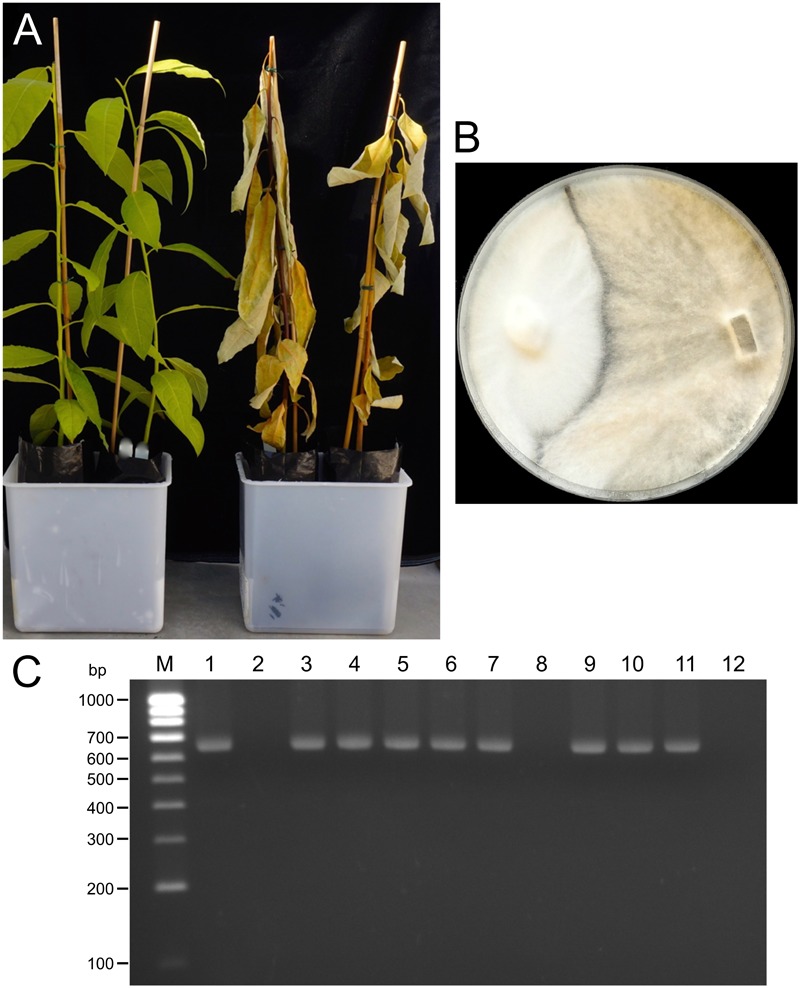
Symptoms on 4-month old avocado plants inoculated with *Entoleuca* sp. (left) and *R. necatrix* (right) **(A)**. Vegetative incompatibility between *Entoleuca* sp. isolates E97-14 (left) and E112-4 (right) grown on PDA **(B)**. RT-PCR detection of EnHV1 in several *Entoleuca* sp. and *R. necatrix* isolates with the primer pair HYPRD-F/HYPRD-R. From left to right: (M) Bioline 100-bp molecular weight ladder, (1–7) *Entoleuca* sp. E97-14, E102-4, E106-14, E107-12, E108-8, E112-4, and E112-8, and (8–12) *R. necatrix* isolates Rn95-12, Rn95-16, Rn106-11, Rn108-10, and Rn97-11 **(C)**.

RT-PCR using the primer pair HYPRD-F/HYPRD-R allowed the detection of the virus in different fungal isolates of the collection (**Figure 6C**). Amplification products were of the expected size (683 bp). Infection by EnHV1 is frequent: from the set of 39 fungal isolates, 26 harbor the virus, including 7 *R. necatrix* (Rn95-12, Rn106-11, Rn108-10, Rn110-15N, Rn114-4, Rn114-15R, and 108-3-CH2014). Twenty-one out of 26 RT-PCR amplicons were sequenced (GenBank Accession Nos. MF598763–MF598783) and used in the subsequent analyses.

### Genetic Variability and Recombination Analysis in EnHV1 Strains

**FIGURE 7:**
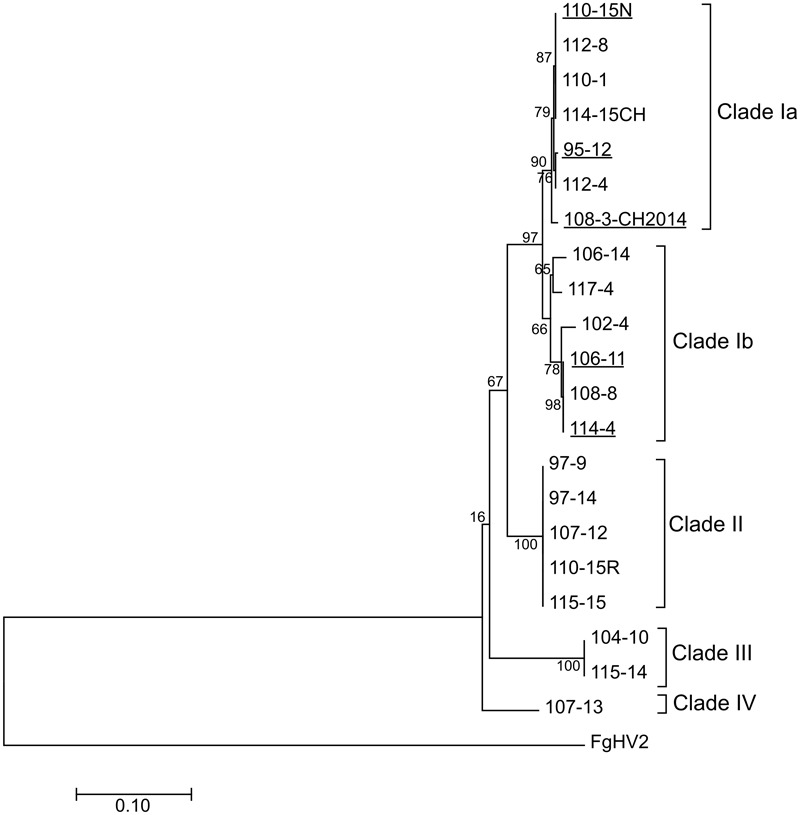
ML phylogenetic tree of the (partial) replicase genomic regions from Entoleuca hypovirus 1. Strains from *R. necatrix* are underlined. The homologous genomic region of FgHV2 was used as out-group. The percentage of trees in which the associated taxa clustered together is shown next to the branches. The tree is drawn to scale, with branch lengths measured in the number of substitutions per site. The analysis involved nucleotide sequences of 21 strains.

Four clades are significantly supported from the phylogenetic analysis of a set of 21 EnHV1 strains (**Figure 7**). Members from *Entoleuca* sp. or *R. necatrix* group either in clades Ia or Ib that include most of the strains, so there is no grouping according to the fungal host. Clade II consists of a sequence shared by five strains, clade III is represented by a sequence shared by two strains, while clade IV has only one member. The highest genetic distance between pairs of strains was 0.144 corresponding to strains 102-4 (clade Ib) and 104-10 (clade III) followed by the distance of 0.142 between strains 104-10 and 107-13 (clades III and IV). The average genetic diversity of the EnHV1 strains was found to be 0.060 ± 0.006. Regarding the selective pressures, the *d*NS/*d*S ratio resulted 0.091, indicating a purifying selection. Tajima’s *D*, and Fu and Li’s *D*^∗^ and *F*^∗^ neutrality test were used to study the evolutionary processes in the RdRp region of the 21 EnHV1 isolates (**Table 1**). These statistics did not show a significant deviation from neutrality, confirming a purifying selection in the EnHV1 strains from avocado-infecting fungi in Spain. Using the SLAC at *P* < 0.1 17 sites under negative pressure were identified in this genomic region. Less conservative tests such as FEL and IFEL showed 51 and 22 negative selected sites, respectively (*P* < 0.1). The FEL test showed one single site under positive pressure at *P* < 0.1 and none at *P* < 0.05. Recombination analysis within the genomic region considered in this work showed two possible recombination events. One recombinant event is predicted for strain 97-9, which seems to have originated from stains 108-3 and 115-14 as major and minor parent, respectively, though it is supported only by two of the nine methods available in RDP4. This same recombination event appeared in other four EnHV1 strains. The other recombinant event is predicted for 107-13 strain coming from strains 114-4 and 115-14 as major and minor parent, respectively, being supported by three methods (**Supplementary Table S1**). GARD analysis using AIC_c_ score resulted in a possible breakpoint at position 501 (LHS *P*-value = 0.038). There is no correlation between the origin of the strains and the genetic identity (for details on the location of the trees see Arjona-Girona and López-Herrera, 2018). Besides, in each orchard and even in each tree, strains can belong to different clades. Some strains from the same tree belong to a different clade (e.g., 110-1 and 110-15 or 114-4 and 114-15CH). Conversely, strains belonging to the same clade were isolated from fungi obtained in trees from different orchards (e.g., 104-10 and 115-14 or 97-9 and 115-15, which shared identical sequences).

**Table 1:** Genetic diversity parameters and neutrality tests^a^ in the population of isolates of *Entoleuca* hypovirus 1.

*d*S ± SE	*d*NS ± SE	*S*	*p*s	Θ	π	*D*	Fu and Li’s *D*^∗^	Fu and Li’s *F*^∗^
0.176 ± 0.05	0.016 ± 0.00	120	0.1942	0.0539	0.0557	0.1227	0.3099	0.2954

*^*a*^*d*S, synonymous substitutions per site (Pamilo, Bianchi, and Li); *d*NS, non-synonymous substitutions per site (Pamilo, Bianchi, and Li); SE, standard error with 1,000 bootstrap replicates; *S*, number of segregating sites; *p*s, *S*/*n*; Θ, population mutation rate; π, nucleotide diversity; *D*, Tajima’s test statistic.*

### Effect of Hypovirus Infection on *Entoleuca* sp.– and *R. necatrix*–Host Interaction

In this study, no *Entoleuca* sp. isolate showed pathogenicity in avocado, regardless of the presence of EnHV1. In contrast, all *R. necatrix* isolates were found to be pathogenic regardless of the presence of the hypovirus (**Figure 6A**). It was not possible to determine the specific phenotypic effect of the hypovirus on the fungal aspect or growth development of *Entoleuca* sp. due to the multiple virus infection status of the isolates. All the curation attempts of EnHV1 have failed so far, and therefore it has not been possible to evaluate a given fungal isolate with or without EnHV1 infection. In one case, after five serial passages and hyphal tip isolations in OA-cycloheximide 300 μg/ml, one E97-14-derived mycelium tested negative in conventional RT-PCR for EnHV1. However, RT-qPCR showed that the virus was still present but at lower titters (not shown). This mycelium showed similar aspect and growth to the original isolate in PDA, but after re-cultivation in PDA EnHV1 titters reached usual Cq values, indicating that the antiviral compound was able to reduce virus concentration. Cycloheximide but not ribavirin delayed *Entoleuca* sp. mycelia growth.

## Discussion

The *Entoleuca* sp. isolates E97-14 and E112-4 are vegetatively incompatible with each other (**Figure 6B**) and their respective EnHV1 strains resulted ascribed to different clades in the virus population object of this study (**Figure 7**). The availability of two complete genomes of EnHV1 allowed their comparison with other members of family *Hypoviridae*, allowing us to include the new virus species in that family. The genome organization of EnHV1 included two ORFs comparable with other members of the newly proposed genus Alphahypovirus such as the type species CHV1 or CHV2 but not others like FgHV1, FgHV2, or SsHV2 that show one single ORF in their genomes (Li et al., 2015). As most of the mycoviruses, the absence of a guanine residue at the start of the 5′-UTR indicates that the RNA is not capped. The IRES located in the 5′-UTR region of the EnHV1 RNA provides a mechanism of translation initiation alternative to the most frequent 5′-cap-dependent ribosome scanning mechanism. In addition, the presence of uORF, also found in other hypoviruses, may enhance translation in specific conditions (Rae et al., 1989; Linder-Basso et al., 2005). Comparative analysis of known IRES in some cellular mRNAs identified a common structural motif forming a Y-type stem–loop structure followed by the AUG triplet or followed by additional stem–loop structures and the AUG triplet. There are no clear proofs of a coupled termination-reinitiation allowing downstream cistron translation for EnHV1, but some clues suggest this possibility. The *Hypoviridae*-type member, CHV1, showed a coupled translation depending on complementary level between the 18S rRNA and the sequence upstream the *UAA*UG (Guo et al., 2009). In this pentanucleotide, the ORF A stop codon and the ORF B start codon overlap, belonging to two different frames. In some victoriviruses, the two cistrons overlap either in a pentanucleotide (Chiba et al., 2013) or in a tetranucleotide, *AUG*A (Hua et al., 2011), and the coupled translation can be enhanced by the presence upstream and downstream these motifs of H-type pseudoknots and hairpin structures, respectively. In the case of EnHV1, there is no overlapping between the ORFs, but the distance is less than 40 nt, thus a coupled translation should not be compromised (Powell, 2010). In addition, a reinitiation of translation could be promoted by the H-type pseudoknot and hairpin structures predicted proximal to ORF2 AUG. Therefore, the hypothesis of coupled termination-reinitiation needs to be supported by further studies as well as the subgenomic RNA accumulation pattern corresponding to the ORF2. The putative ORF1 lacks of similarity to other protein sequences in the databases while there are no predicted functional domains in the deduced aa sequence, such as the papain-like proteinase domain in CHV1 (Choi et al., 1991). In other hypoviruses, the peptidase domain locates in the ORF2 that encodes a polyprotein that includes the RdRp domain, such as in FgHV1 (Wang et al., 2013). In the case of EnHV1, ORF1 or ORF2 apparently lack of any peptidase domains. Thus, we cannot assign any function to the ORF1 in this virus species on basis to bioinformatic analysis. EnHV1 ORF2 shows one domain matching to helicases and another region that shows homology to RdRp domains, allowing us to assign a replicase role to the protein putatively encoded by this ORF. The comparison of the two available genomes of EnHV1 showed the highest conservation in the 5′-UTR and the genomic regions encoding for the helicase (11,424–12,240) and the putative RdRp (9,612–9,934) domains in the replicase. Other highly conserved regions may encode for other critical functions of the polyprotein or reflect structural stringencies of the RNA that limit sequence changes.

Antiviral activity of *Entoleuca* sp. was investigated by analyzing the EnHV1-derived vsiRNAs. VsiRNA populations were well distributed along the genome but clustered preferentially at the 5′-UTR and ORF1 regions for all the 20-nt, the 21-nt, the 22-nt, and the 24-nt populations indicating similar target affinities among Dicer-like (DCL) enzymes (Donaire et al., 2009). The predominant representation of the 20- and 21-nt class and the prevalence of A/U at the 5′- and 3′-positions of the vsiRNAs derived from EnHV1 were equivalent to those for several *R. necatrix* mycoviruses (Yaegashi et al., 2016), suggesting the presence of homologous silencing genes and mechanisms in *Entoleuca* sp. Remarkably, reads shorter than 21 nt can be considered as vsiRNAs given that aligned to EnHV1. VsiRNAs smaller than 21 nt have been observed in the fungal species *Heterobasidion annosum* and resulted useful for virus diagnosis (Vainio et al., 2015). On the other hand, negative-sense vsiRNAs prevailed over positive ones in all populations aligning to EnHV1. This is in contrast with most plant virus examples (Donaire et al., 2009; Velasco et al., 2015; Cretazzo and Velasco, 2017).

An RT-PCR test allowed the determination of EnHV1 in a high proportion of isolates in a collection of 39 fungi recovered from avocado roots (67%). Phylogenetic analysis showed that 13 of the 21 strains analyzed group into clade I that includes members from *Entoleuc*a sp. and *R. necatrix* (**Figure 7**). Given that EnHV1 strains from *R. necatrix* are underrepresented in this analysis, a wider population study would probably identify strains from this host also grouping in clades II, III, or IV. The average nucleotide diversity (π) of the EnHV1 population was found to be 0.056, in a range similar to those described in genes of widely distributed plant viruses (Pesqueira et al., 2016; Velasco et al., 2016) and comparable to the mycovirus Heterobasidion RNA virus 6 (HetRV6), where the authors report nucleotide diversities within a range of 0.019–0.067 for the partial replicase gene in different populations of the virus (Vainio et al., 2012). According to the neutrality tests performed, there is a purifying selection in the EnHV1 strains collected from avocado-infecting fungi in Spain. Vainio et al. (2015) reported a *d*NS/*d*S ratio of 0.054 for a gene of HetRV6, supporting a purifying selection in this mycovirus as well. Given that there are no founder events in the EnHV1 strains, it is plausible that multiple entries of EnHV1-infected fungi have occurred in the avocado orchards in Spain. It can also be assumed that the fungal hosts and their viruses were already present in today’s orchards before avocado was grown in the area, as *R. necatrix* infects many different crops including almonds, olive trees, and vines (López-Herrera and Zea-Bonilla, 2007). Recombination events in the sequences considered in this work resulted probable, although the evidence supporting this was low. A longer portion of the regions studied or extending the analysis to more genomic regions in additional virus strains may help in clarifying the role played by recombination in EnHV1 evolution. Both the presence of EnHV1 in different fungal isolates in the same orchards and the lack of correlation between EnHV1 strain clustering and fungal species source suggest intraspecific and interspecific exchange of the virus, notwithstanding the vegetative incompatibility among fungal strains and species. While this manuscript was still in revision, Arjona-López et al. (2018) reported the partial sequence of another strain of EnHV1 from a *R. necatrix* isolate belonging to the same collection of fungal isolates described in this work. That virus species was named *Rosellinia necatrix* hypovirus 2 (RnHV2, LC333733).

An RT-qPCR test allowed EnHV1 detection at low titters given that conventional RT-PCR provided negative results in some apparently cured isolates that after re-cultivation tested positive. Similarly to other mycovirus works (Romo et al., 2007; Aoki et al., 2009; Cai et al., 2012), attempts to cure *Entoleuca* sp. was unsuccessful and consequently it has been impossible to definitely explore the mycovirus–host interaction object of this study. Moreover, each fungal isolate studied in the collection has shown a complex virome composition (Velasco et al., unpublished), making it difficult to compare a given fungal isolate both virus-free and infected only by EnHV1. These difficulties in obtaining virus-free isolates can eventually be overcome by generating protoplasts (Wang et al., 2013). Besides, the development of an infectious clone combined with protoplast fusion would allow testing the transfection to *Entoleuca* sp. or *R. necatrix* (Yaegashi et al., 2012; Chiba et al., 2013). In spite of that, biological assays have provided a consistent hint: all the *Entoleuca* sp. isolates in this collection resulted hypovirulent to avocado, while all the *R. necatrix* isolates studied showed to be pathogenic, regardless of the hypovirus infection. Finally, further studies are advised to explore additional hypovirulence sources in the pathosystem *R. necatrix*-avocado.

## Author Contributions

LV and CL-H designed the research. LV, IA-G, and MA-F performed the experiments. LV and EC did the bioinformatic analysis. CL-H managed the fungal collection. LV, EC, and CL-H wrote the manuscript. All authors read and approved the manuscript.

## Conflict of Interest Statement

The authors declare that the research was conducted in the absence of any commercial or financial relationships that could be construed as a potential conflict of interest.

## Footnotes

1 http://regrna2.mbc.nctu.edu.tw

2 http://www.datamonkey.org

## Supplementary Material

FIGURE S1 Relative abundances of small RNA reads from 17 to 32 nt obtained from *Entoleuca* sp. isolate E97-14. Black and gray bars indicate the populations of small RNAs and the specific vsiRNAs of EnHV1 strain 97-14, respectively.

TABLE S1 Recombination events detected in Entoleuca hypovirus 1 strains by different recombination algorithms implemented in RDP4.
