# Supplementary figures and images for: A Novel Hypovirus Species From Xylariaceae Fungi Infecting Avocado
Source: Front Microbiol. 2018 May 8;9:778. doi: 10.3389/fmicb.2018.00778 (PMC5952064; doi:10.3389/fmicb.2018.00778)

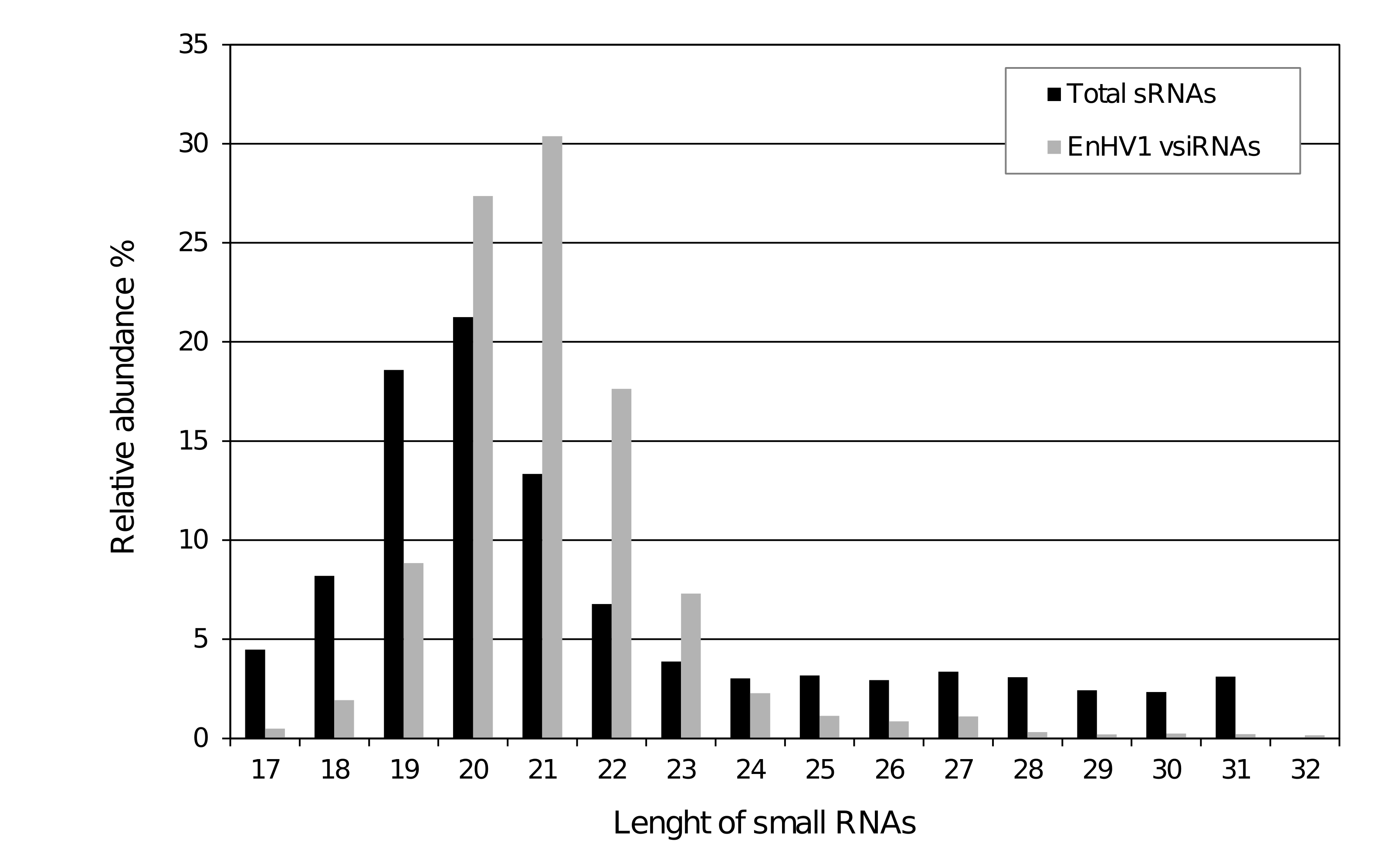

Supplement: FIGURE S1 — Relative abundances of small RNA reads from 17 to 32 nt obtained from Entoleuca sp. isolate E97-14. Black and gray bars indicate the populations of small RNAs and the specific vsiRNAs of EnHV1 strain 97-14, respectively. [file Image_1.TIF]
