# Supplementary material for: A Novel Hypovirus Species From Xylariaceae Fungi Infecting Avocado
Source: Front Microbiol. 2018 May 8;9:778. doi: 10.3389/fmicb.2018.00778 (PMC5952064; doi:10.3389/fmicb.2018.00778)
Supplement: TABLE S1 — Recombination events detected in Entoleuca hypovirus 1 strains by different recombination algorithms implemented in RDP4. [file Table_1.docx]

**Supplementary Table S1.** Recombination events detected in Entoleuca hypovirus 1 strains by different recombination algorithms implemented in RDP4.

|  |  |  | Inference algorithm (*P*-value) | | | | | | | |  |
| --- | --- | --- | --- | --- | --- | --- | --- | --- | --- | --- | --- |
| Recombinant | Major parent | Minor parent | GENECONV | BOOTSCAN | MAXCHI | CHIMERA | SISCAN | 3SEQ | LARD | RDP | Recombination breakpoint^a^ |
| 107-13 | 114-4 | 115-14 | - | - | 1.96 x 10^-3^ | 6.92 x 10^-3^ | 9.96 x 10^-2^ | - | - | - | 358-471 |
| 97-9 | 108-3-CH2014 | 115-14 | - | - | 2.16 x 10^-2^ | - | 2.02 x 10^-5^ | - | - | - | 154-464 |
| 97-14 | 108-3-CH2014 | 115-14 | - | - | 2.16 x 10^-2^ | - | 2.02 x 10^-5^ | - | - | - | 11,080-11,390 |
| 107-12 | 108-3-CH2014 | 115-14 | - | - | 2.16 x 10^-2^ | - | 2.02 x 10^-5^ | - | - | - | 154-464 |
| 110-15R | 108-3-CH2014 | 115-14 | - | - | 2.16 x 10^-2^ | - | 2.02 x 10^-5^ | - | - | - | 154-464 |
| 115-15 | 108-3-CH2014 | 115-14 | - | - | 6.28 x 10^-3^ | - | 2.02 x 10^-5^ | - | - | - | 154-464 |

^a^Recombination breakpoints in the partial ORF 2 sequences corresponding to Accession nos. MF598776, MF598778, MF598779, MF598780 and MF598781 or in the complete genome of EnHV1 97-14 (MF536690).
